# Ceacam1 Separates Graft-versus-Host-Disease from Graft-versus-Tumor Activity after Experimental Allogeneic Bone Marrow Transplantation

**DOI:** 10.1371/journal.pone.0021611

**Published:** 2011-07-06

**Authors:** Sydney X. Lu, Lucy W. Kappel, Anne-Marie Charbonneau-Allard, Renée Atallah, Amanda M. Holland, Claire Turbide, Vanessa M. Hubbard, Jimmy A. Rotolo, Marsinay Smith, David Suh, Christopher King, Uttam K. Rao, Nury Yim, Johanne L. Bautista, Robert R. Jenq, Olaf Penack, Il-Kang Na, Chen Liu, George Murphy, Onder Alpdogan, Richard S. Blumberg, Fernando Macian, Kathryn V. Holmes, Nicole Beauchemin, Marcel R. M. van den Brink

**Affiliations:** 1 Department of Immunology and Medicine, Memorial Sloan-Kettering Cancer Center, New York, New York, United States of America; 2 Department of Immunology, Weill Cornell Graduate School of Medical Sciences, New York, New York, United States of America; 3 Goodman Cancer Center and Departments of Biochemistry, Medicine, and Oncology, McGill Cancer Center, Montreal, Quebec, Canada; 4 Department of Pathology, Albert Einstein College of Medicine, Bronx, New York, United States of America; 5 Department of Molecular Pharmacology and Chemistry, Memorial Sloan-Kettering Cancer Center, New York, New York, United States of America; 6 Department of Hematology and Oncology, Charité CBF - Universitätsmedizin Berlin, Berlin, Germany; 7 Department of Pathology, University of Florida College of Medicine, Gainesville, Florida, United States of America; 8 Department of Pathology, Brigham and Women's Hospital, Boston, Massachusetts, United States of America; 9 Department of Medicine, Yale University Medical School, New Haven, Connecticut, United States of America; 10 Department of Gastroenterology, Brigham and Women's Hospital, Boston, Massachusetts, United States of America; 11 Department of Microbiology, University of Colorado School of Medicine, Aurora, Colorado, United States of America; Universidade de Sao Paulo, Brazil

## Abstract

**Background:**

Allogeneic bone marrow transplantation (allo-BMT) is a potentially curative therapy for a variety of hematologic diseases, but benefits, including graft-versus-tumor (GVT) activity are limited by graft-versus-host-disease (GVHD). Carcinoembryonic antigen related cell adhesion molecule 1 (Ceacam1) is a transmembrane glycoprotein found on epithelium, T cells, and many tumors. It regulates a variety of physiologic and pathological processes such as tumor biology, leukocyte activation, and energy homeostasis. Previous studies suggest that Ceacam1 negatively regulates inflammation in inflammatory bowel disease models.

**Methods:**

We studied Ceacam1 as a regulator of GVHD and GVT after allogeneic bone marrow transplantation (allo-BMT) in mouse models. *In vivo*, Ceacam1^−/−^ T cells caused increased GVHD mortality and GVHD of the colon, and greater numbers of donor T cells were positive for activation markers (CD25^hi^, CD62L^lo^). Additionally, Ceacam1^−/−^ CD8 T cells had greater expression of the gut-trafficking integrin α_4_β_7_, though both CD4 and CD8 T cells were found increased numbers in the gut post-transplant. Ceacam1^−/−^ recipients also experienced increased GVHD mortality and GVHD of the colon, and alloreactive T cells displayed increased activation. Additionally, Ceacam1^−/−^ mice had increased mortality and decreased numbers of regenerating small intestinal crypts upon radiation exposure. Conversely, Ceacam1-overexpressing T cells caused attenuated target-organ and systemic GVHD, which correlated with decreased donor T cell numbers in target tissues, and mortality. Finally, graft-versus-tumor survival in a Ceacam1^+^ lymphoma model was improved in animals receiving Ceacam1^−/−^ vs. control T cells.

**Conclusions:**

We conclude that Ceacam1 regulates T cell activation, GVHD target organ damage, and numbers of donor T cells in lymphoid organs and GVHD target tissues. In recipients of allo-BMT, Ceacam1 may also regulate tissue radiosensitivity. Because of its expression on both the donor graft and host tissues, this suggests that targeting Ceacam1 may represent a potent strategy for the regulation of GVHD and GVT after allogeneic transplantation.

## Introduction

Ceacam1 is a member of a large family of carcinoembryonic antigen proteins [2]. It is primarily a type I transmembrane protein with multiple splice variants [9], though soluble forms also exist. Ceacam1 is widely expressed on a variety of tissues including endothelium [10], epithelium [11], hematopoietic cells [12] and both hematologic and solid tumors, and interacts in a homophilic and heterophilic fashion with physiological and pathogen-associated ligands, including carcinoembryonic antigen and the *Neisseria spp.* proteins [13].

Some Ceacam1 isoforms contain intracellular ITIM motifs, and activation of Ceacam1 results in the recruitment of the SHP-1 and SHP-2 phosphatases [8], [14], which dephosphorylate substrates across a range of signaling pathways. Ceacam1 thus inhibits T cell receptor (TCR) signaling and suppresses multiple aspects of T cell function. Ceacam1 agonists attenuate cytokine secretion, T cell polarization and cytolytic function. *In vivo*, ligation of Ceacam1 with soluble ligands or over-expression of ITIM-containing Ceacam1 isoforms on T cells attenuates experimental colitis [7], [8]. Additionally, Ceacam1 is also expressed on intestinal T cells in patients with Celiac disease [15] and ulcerative colitis [16], and may represent an attempt by the immune system to negatively regulate these inflammatory processes.

In addition to immune regulation, Ceacam1 exerts a wide variety of other biological functions. It is a cell-cell adhesion molecule [17], [18], and a receptor for a variety of commensal and pathogenic microbes in mouse and man [17], [19], [20], [21]. Ceacam1 also regulates angiogenesis [6], energy homeostasis [22], and tumor biology [23], [24], [25]. Ceacam1 regulates the tumorigenesis of colon cancers, and is a prognostic factor in lung adenocarcinoma. Tumor expression of Ceacam1 may regulate tumor angiogenesis and invasion, and the expression of both Ceacam1 and CEA by tumors may inhibit the functions of tumor infiltrating lymphocytes.

Allo-BMT is an established therapy with curative intent for a variety of hematologic malignancies and non-malignant conditions [26]. Alloreactive T cells of donor origin play a criticial role in both GVHD, a major complication of allo-BMT, and graft-versus-tumor activity, a major contributor to the efficacy of allo-BMT as a cancer therapy.

Donor-recipient antigenic disparity, donor T cells, and tissue injury resulting in inflammation due to the conditioning regimen all contribute to GVHD, which primarily affects intestines, liver, skin and thymus [27].

Ceacam1 is expressed both on leukocytes (especially T cells), as well as on epithelial and endothelial cells, which are prominent components of the parenchyma of the above-mentioned GVHD target organs. In addition, Ceacam1 is upregulated on many tumors. In this report, we assess the impact of Ceacam1 on alloreactive T cells in the donor allograft, as well as the effects of Ceacam1 deficiency on recipients of allo-BMT with respect to GVHD and GVT activity.

## Results

### Ceacam1 on donor T cells and recipient tissues can regulate GVHD mortality

We assessed Ceacam1 regulation of GHVD on donor T cells or recipients in two well-described major histocompatibility complex (MHC) class I/II-disparate models C57BL/6 (B6, H-2^b^)→BALB/c (H-2^d^) and BALB/c→B10.BR (H-2^k^). We used Ceacam1^−/−^ B6 mice [28], Ceacam1-transgenic (Tg) B6 mice (described in **Figure S1**), and Ceacam1^−/−^ BALB/c mice [28] as the source of donor T cells or recipients. In all experiments, recipients received split-dose lethal irradiation (BALB/c: 8.5 Gy, B10.BR: 11 Gy) and a graft of 5×10^6^ allogeneic T cell depleted bone marrow (TCD-BM) of wildtype (WT) origin, with or without splenic T cells.

**Figure 1:**
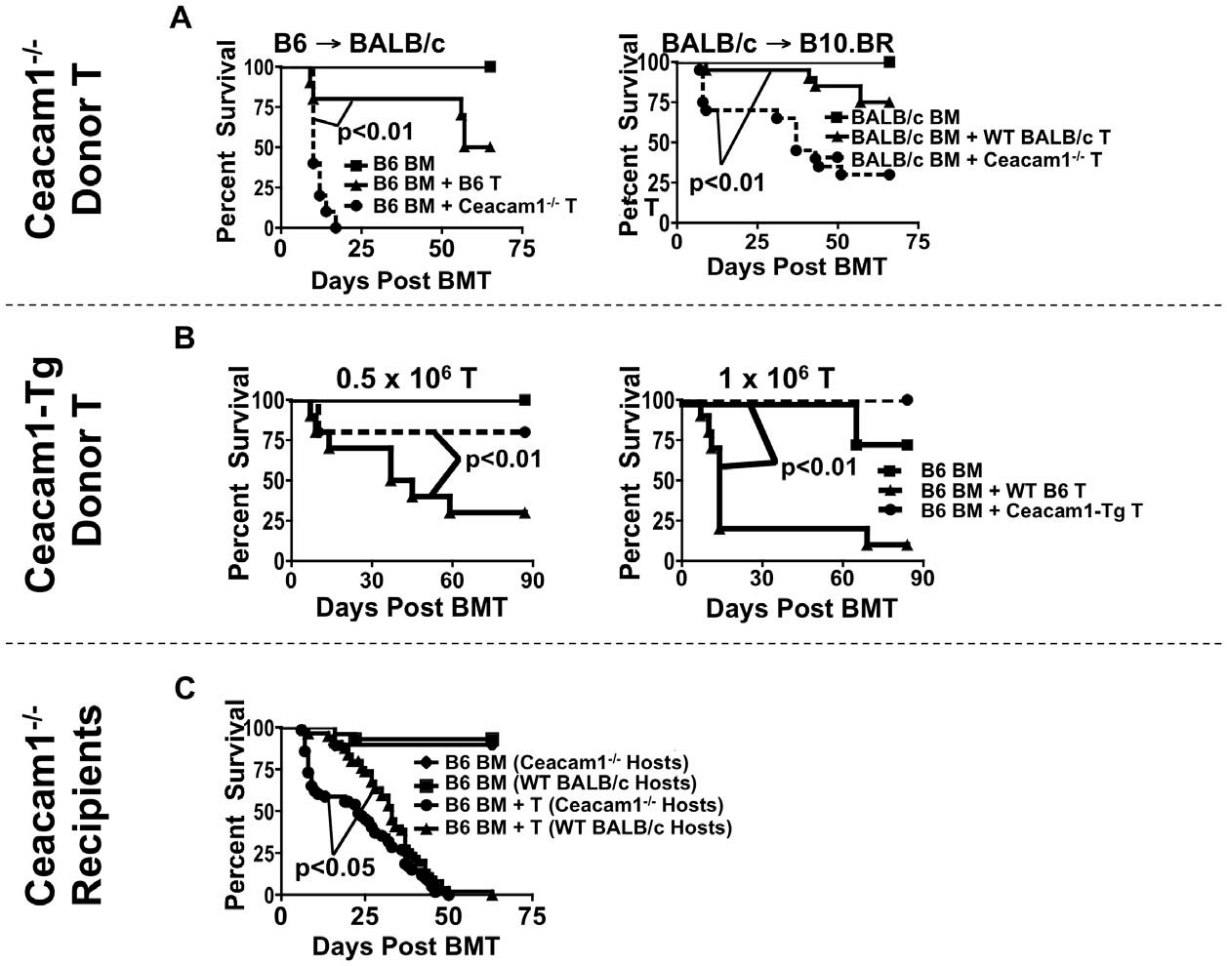
Ceacam1 regulates GVHD mortality. A) LEFT: 5×10^6^ TCD-BM ± 1×10^6^ T→BALB/c. N = 10/group. Representative data from one of four experiments. RIGHT: 5×10^6^ TCD-BM ± 2×10^6^ T→B10.BR. N = 20/group combined from two experiments. Square: BM only. Triangle: WT T. Circle: Ceacam1^−/−^ T. B) LEFT: 5×10^6^ TCD-BM ± 0.5×10^6^ T→BALB/c. N = 10/group. Representative data from one of three experiments. RIGHT: 5×10^6^ TCD-BM ± 1×10^6^ T→BALB/c. N = 10/group. Representative data from one of two experiments. Square: BM only. Triangle: WT T. Circle: Ceacam1-Tg T. C) 5×10^6^ TCD-BM ± 1×10^6^ T→BALB/c. Combined N = 55 in groups receiving T cells from six experiments. Diamond: Ceacam1^−/−^ recipients, BM only. Square: WT recipients, BM only. Triangle: WT recipients, BM+T. Circle: Ceacam1^−/−^ recipients, BM+T.

We first transplanted irradiated BALB/c mice with B6 TCD-BM with WT or Ceacam1^−/−^ T cells, and observed that recipients of Ceacam1^−/−^ T cells had significantly increased mortality compared to recipients of WT T cells (**Figure 1A**, left). We confirmed this in a second MHC-disparate allo-BMT model, BALB/c→B10.BR (**Figure 1A**, right).

We next asked whether T cells overexpressing Ceacam1 would cause less disease, and transplanted BALB/c recipients with 0.5×10^6^ or 1×10^6^ donor WT or Ceacam 1-Tg T cells. At both doses, recipients of Ceacam1-Tg T cells showed attenuated mortality (**Figure 1B**).

Finally, we assessed the role of Ceacam1 on tissues of allo-BMT recipients, and transferred TCD-BM+T cells into WT vs. Ceacam1^−/−^ BALB/c recipients. This revealed that Ceacam1^−/−^ recipients had increased early (but not overall) mortality, with nearly 50% of mice succumbing within the first week (**Figure 1C**).

### Ceacam1 is an important regulator of GVHD target organ damage

We next asked whether Ceacam1 regulated GVHD target organ damage, and again assessed effects of Ceacam1 deficiency or overexpression on donor T cells, and Ceacam1^−/−^ allo-BMT recipients.

**Figure 2:**
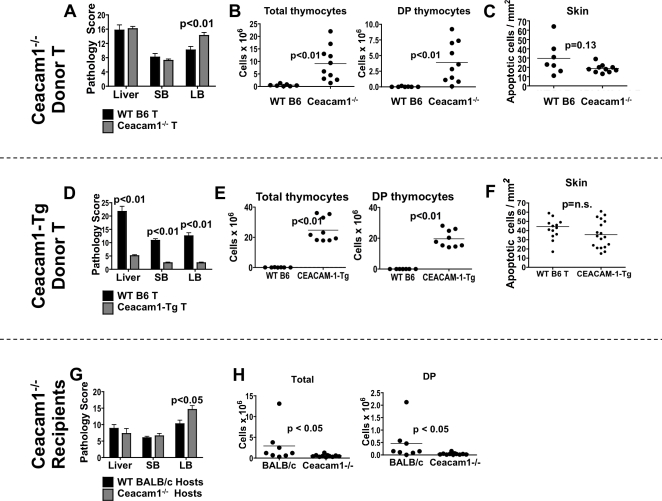
Ceacam1 regulates GVHD target organ damage. A) 5×10^6^ TCD-BM ± 1×10^6^ T→BALB/c. Day 21. Histopathology score. N = 5–12/group. SB, small bowel. LB, large bowel. B) Thymocyte count and apoptotic skin cells from (A). N≥7/group. Day 21. One of two representative experiments. C) Apoptotic skin cells from (A). N≥7/group. Day 21. D) 5×10^6^ TCD-BM ± 1×10^6^ T→BALB/c. Histopathology score. Day 21. N≥7/group. SB, small bowel. LB, large bowel. E) Thymocyte counts from (E): N≥7/group. One of two independent experiments. DP: CD4^+^CD8^+^ double-positive thymocytes. Day 21. F) Apoptotic skin cells from (E). N≥7/group. Day 21. G) 5×10^6^ TCD-BM ± 1×10^6^ T→WT or Ceacam1^−/−^ BALB/c. Histopathology score. Day 14. N≥12/group. SB, small bowel. LB, large bowel. H) Thymocyte count from (I). N≥8/group. One of two independent experiments. DP: CD4^+^CD8^+^ double-positive thymocytes. Day 14.

We observed that recipients of Ceacam1^−/−^ T cells had more severe large intestinal GVHD (**Figure 2A**). Surprisingly however, these mice exhibited less thymic GVHD, as determined by thymic cellularity and numbers of CD4^+^CD8^+^ double-positive (DP) thymocytes (**Figure 2**). Thymic cellularity from age/sex-matched non-transplanted animals are shown in **Figure S2**. We also observed a modest trend towards less skin GVHD in recipients of Ceacam1^−/−^ T cells (**Figure 2C**), suggesting that Ceacam1^−/−^ T cells caused preferential damage to the (large) intestines.

In experiments comparing recipients of WT and Ceacam1-Tg T cells on day 21 post-transplant, we found that recipients of Ceacam1-Tg T cells demonstrated significantly less GVHD of the liver, intestines, and thymus compared to recipients of WT T cells, but similar skin GVHD (**Figures 2D–F**). This appears to suggest that Ceacam1-Tg T cells caused less GVHD overall, with relatively little organ specificity.

Finally, we assessed Ceacam1^−/−^ allo-BMT recipients on day 14 post-transplant. In correspondence with increased early GVHD mortality, Ceacam1^−/−^ allo-BMT recipients showed increased large bowel damage and thymic GVHD (**Figure 2G–H**).

### Ceacam1 regulates donor T cell numbers in lymphoid tissues and target organs during GVHD

We next assessed the numbers of donor CD4 and CD8 effector T cells after transfer of Ceacam1^−/−^ or Ceacam1-Tg T cell-containing allografts, or in Ceacam1^−/−^ allo-BMT recipients.

**Figure 3:**
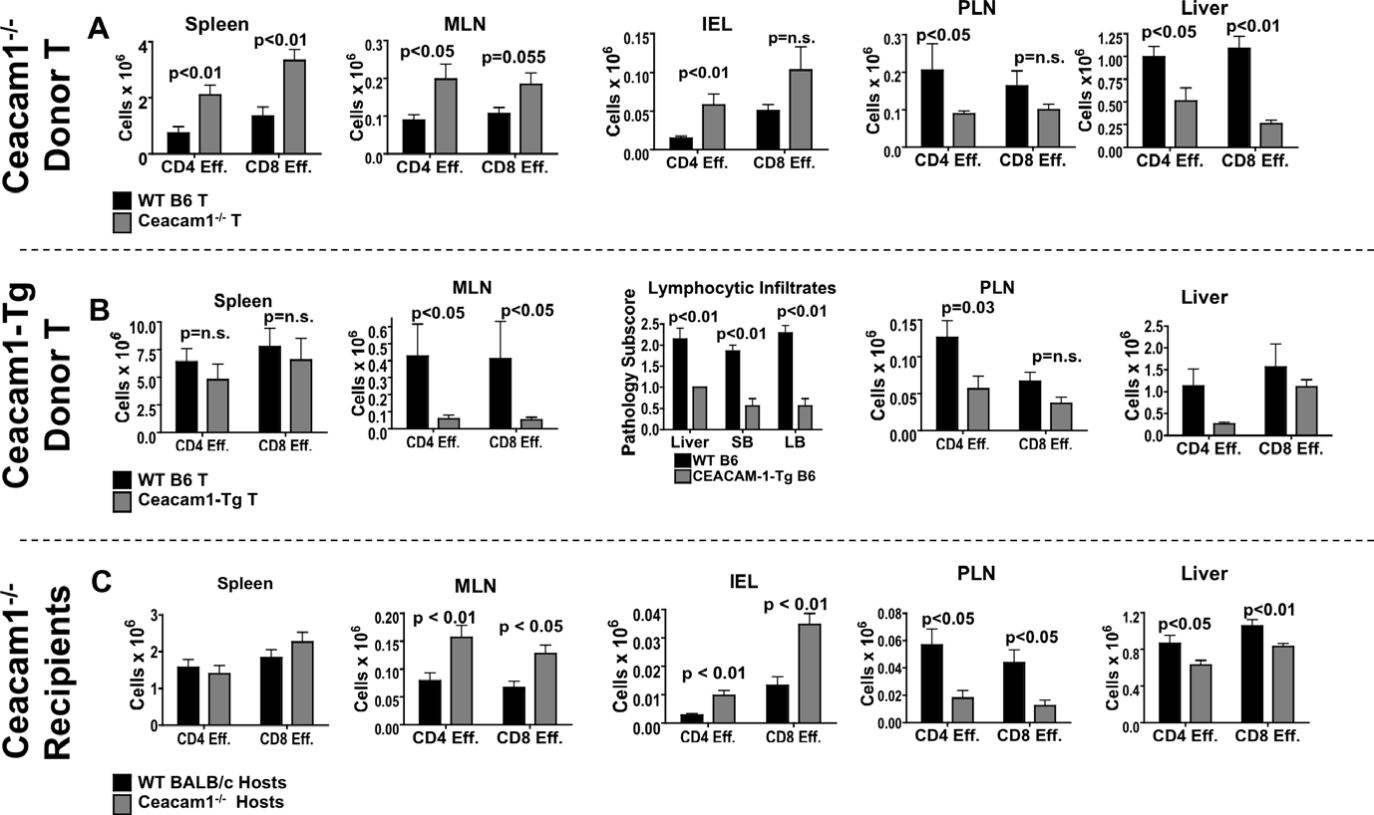
Ceacam1 regulates donor T cell numbers in GVHD target tissues. A) 5×10^6^ TCD-BM ± 1×10^6^ T→BALB/c. Day 14. Donor CD44^+^CD62L^−^ effector cells were enumerated. Donor T cells are shown. N = 5–18/group. Combined data from two to three experiments. B) 5×10^6^ TCD-BM ± 1×10^6^ T→BALB/c. Day 14. Donor CD44^+^CD62L^−^ effector cells were enumerated. N = 7 group. Combined data from two experiments. C) 5×10^6^ TCD-BM ± 1×10^6^ T→BALB/c. Day 14. Donor CD44^+^CD62L^−^ effector cells were enumerated. N = 7–18 group. Combined data from two to four experiments.

Comparing recipients of WT T cells with those receiving Ceacam1^−/−^ T cells, we observed increased numbers of Ceacam1^−/−^ donor alloactivated effector T cells in the spleen, MLN, and IEL of allo-BMT recipients (**Figure 3A**), which was associated with a concomitant decrease in the number of Ceacam1^−/−^ alloactivated CD4 and CD8 T cells in the PLN and liver.

When we analyzed organs of recipients of allografts containing WT vs. Ceacam1-Tg T cells, we noted decreased numbers of donor effector T cells in the MLN, PLN, and liver (**Figure 3B**). Via histopathological analysis, we also observed decreased numbers of total lymphocytic infiltrates into the liver, small and large bowels by histopathology (**Figure 3B**), as well as decreased neutrophilic infiltrates in these organs (data not shown). Finally, we assessed infiltrating T cells in WT and Ceacam1^−/−^ allo-BMT recipients, and observed increased numbers of donor alloactivated effector T cells in the MLN and IEL of Ceacam1^−/−^ allo-BMT recipients, but decreased numbers of these cells in the PLN and liver (**Figure 3C**).

### Ceacam1 regulates the sensitivity of the small intestine to radiation injury

**Figure 4:**
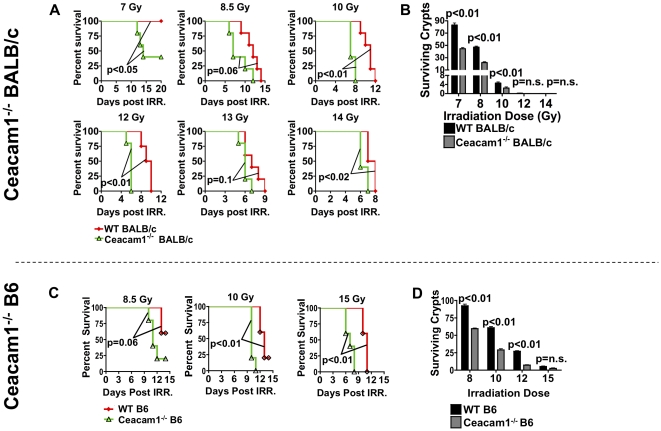
Ceacam1^−/−^ mice exhibit increased radiation sensitivity and intestinal damage from ionizing radiation. A) WT and Ceacam1^−/−^ BALB/c mice were irradiated as a single-dose. N = 5/group. One of two independent experiments. B) Experiment as in A. Quantification of surviving and regenerating crypts from the terminal ileum. N = 3/group, 9 sections per terminal ileum taken. C) WT and Ceacam1^−/−^ B6 mice were irradiated as a single-dose. N = 5/group. One of two independent experiments. D) Experiment as in C. Quantification of surviving and regenerating crypts from the terminal ileum. N = 3/group, 9 sections per terminal ileum taken.

The accelerated early mortality of Ceacam1^−/−^ allo-BMT recipients, together with increased accumulation of donor T cells in GI tract and mesenteric lymph nodes, but decreased numbers peripheral lymph nodes (**Figure 3C**), led us to ask whether Ceacam1 had differential effects in regulating GVHD target organ damage for various target organs and tissues. In the context of Ceacam1^−/−^ recipients, we therefore tested the radiation sensitivity of Ceacam1^−/−^ mice used as hosts, by irradiating WT and Ceacam1^−/−^ BALB/c mice and assessing survival. Ceacam1^−/−^ animals showed increased kinetics of mortality, and in some cases, overall mortality after radiation injury (**Figure 4A**). Similar results were obtained on the B6 background (**Figure 4C**). We then enumerated regenerating and surviving crypts in the small intestine (terminal ileum) at 84 hours after irradiation to assess intestinal radiation damage, and observed that Ceacam1^−/−^ mice had fewer regenerating and surviving crypts as compared with WT counterparts (**Figure 4B and D**), indicating greater damage to the small intestine across a wide range of radiation doses. It is thus quite likely that our radiation-containing conditioning regimen for transplant recipients also contributes in part to their survival kinetics, selective GVHD target organ damage (**Figure 2G–H**), and the selective accumulation of donor T cells in lymphoid tissues and target organs (**Figure 3C**) in these recipients.

### Donor Ceacam1^−/−^ CD8 T cells express higher levels of integrin α_4_β_7_ post-transplant

**Figure 5:**
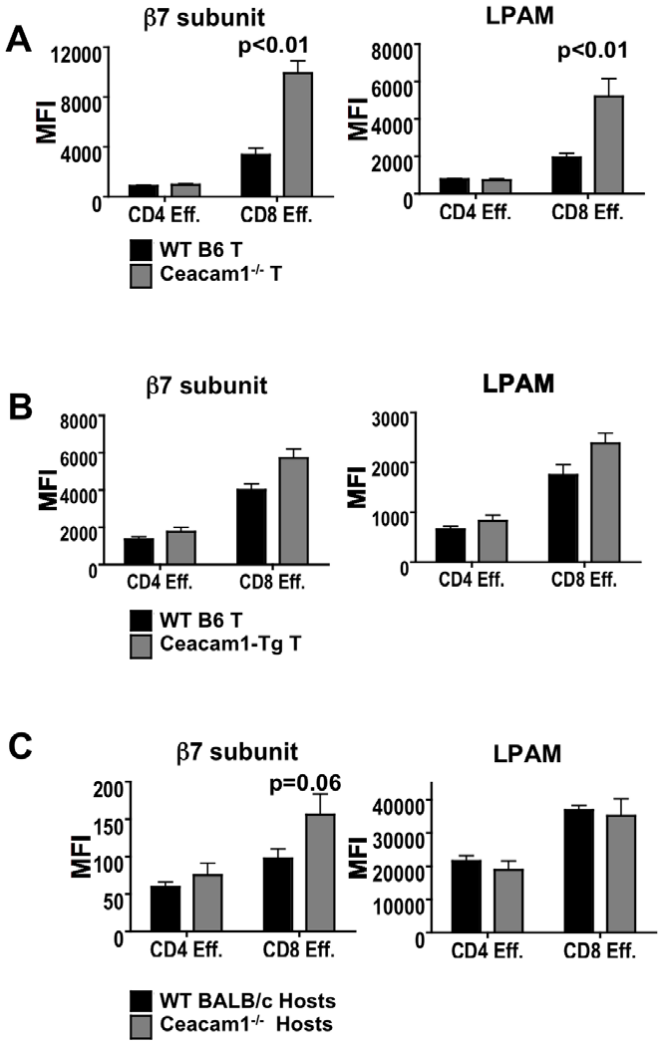
Ceacam1^−/−^ donor CD8 T cells in the spleen express increased levels of integrin β7 subunit post-transplant. A) 5×10^6^ TCD-BM ± 1×10^6^ WT or Ceacam1^−/−^ T→BALB/c. Day 14. N = 6 to 10/group. One of two independent experiments. B) 5×10^6^ TCD-BM ± 1×10^6^ WT or Ceacam1-Tg T→BALB/c. Day 14. N = 6 to 10/group. One of two independent experiments. C) 5×10^6^ TCD-BM ± 1×10^6^ T→WT or Ceacam1^−/−^ BALB/c. Day 14. N = 5 to 8/group. One of two independent experiments.

Next, we studied a variety of possible mechanisms by which Ceacam1 may regulate donor T cell function. We analyzed donor WT and Ceacam1^−/−^ alloactivated splenic T cells on day 14 after allo-BMT for trafficking molecules, and found that Ceacam1^−/−^ CD8^+^ CD44^+^CD62L^−^ effector T cells expressed higher levels of integrin β_7_ subunit and the gut homing integrin α_4_β_7_ (**Figure 5A**), which is important for intestinal GVHD [31], [32], [33]. However, WT vs. Ceacam1−/− CD4 effector T cells had similar integrin β_7_ subunit expression, yet also accumulated in greater numbers in the gut (**Figure 3A**), suggesting that regulation of target organ damage by Ceacam1 is very likely to involve multiple additional mechanisms beyond trafficking molecule expression. Additionally, levels of the α_E_ subunit, which forms integrin α_E_β_7_, were similar, as were levels of CCR9, CD31, PSGL1, CCR7, CXCR3, and LFA1 (data not shown).

When we assessed the expression of trafficking molecules in recipients of WT vs. Ceacam1-Tg T cell allografts, we found no significant differences in levels of β_7_ subunit, integrin α_4_β_7_ (**Figure 5B**), or any other molecules. This is consistent with the systemic reduction in GVHD in recipients of Ceacam1-Tg T cells. Finally, we assessed trafficking molecules in irradiated WT vs. Ceacam1^−/−^ recipients of identical donor allografts, and observed again that donor CD8, but not CD4 splenic T cells in Ceacam1^−/−^ recipients had a trend towards increased expression of the β_7_ subunit, although this was not directly reflected in increased expression of integrin α_4_β_7_. (**Figure 5C**). Taken together, these observations suggest that regulation of trafficking molecule expression by Ceacam1 is only one component of how it regulates GVHD target organ damage.

### Ceacam1 is expressed on T cells during alloactivation

**Figure 6:**
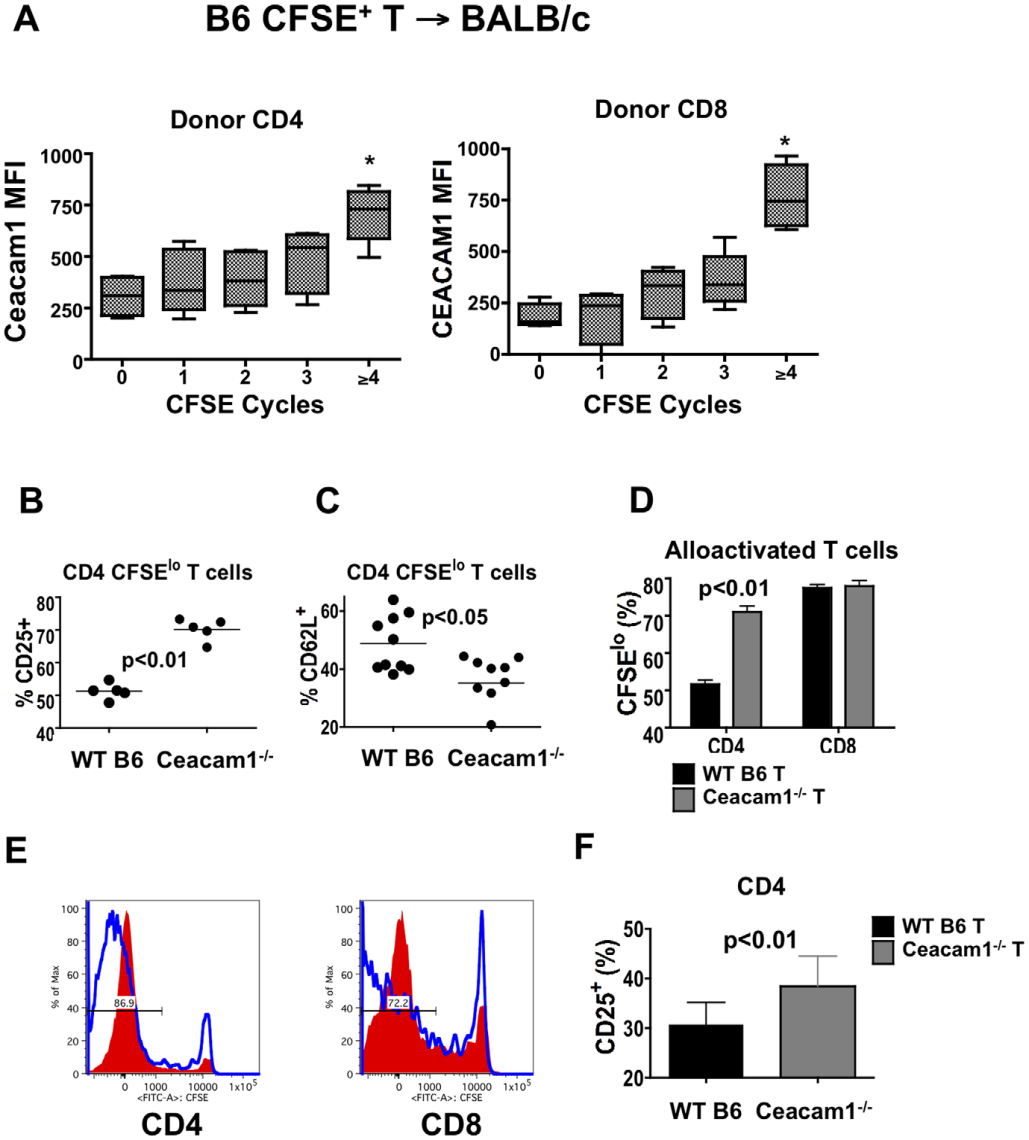
Ceacam1 is expressed transiently upon T cell alloactivation, and regulates T cell activation and proliferation. A) 10^7^ CFSE-labeled B6 or B6 CD45.1 splenic T cells→BALB/c (8.5 Gy). Spleens analyzed day 2. Donor CD4 and CD8 T cells were analyzed by number of divisions viaCFSE dilution. Ceacam1 median fluorescence intensity (MFI) is shown. N = 6, combined from three experiments. * p<0.05 for MFI difference vs. non-dividing cells. B) 10^7^ WT or Ceacam1^−/−^ CFSE-labeled T→BALB/c. Day 3, splenic T cells. Percentage of donor CD25 positive CD4 CFSE^lo^ cells are shown. N = 5–10/group. Combined data from three experiments. C) Percentage of CD62L positive CD4 CFSE^lo^ cells from experiment as in (A). Combined from four experiments, total N = 10/group. D) Percentage of donor CFSE^lo^, fast-proliferating allo-activated T cells from experiment as in (A). N = 5/group, one of three experiments shown. E) 10^7^ CFSE^+^ B6 or CEACAM1-Tg T→BALB/c. Day 3 spleen. CFSE dilution on donor T cells are shown. Shaded: Ceacam1-Tg; open: WT. N = 2/group. Representative data from one of two experiments. F) 10^7^ CFSE^+^ B6 T→WT or Ceacam1^−/−^ BALB/c. Percentage of donor CD25 positive CD4 CFSE^lo^ cells are shown. N = 10/group. Combined data from four experiments.

Ceacam1 can be found on activated T cells [7], [8], [29] and, we thus performed a kinetic analysis of Ceacam1 expression on T cells during alloactivation. We adoptively transferred CFSE-labeled B6 T cells into irradiated BALB/c recipients, and observed transient expression only on day 2 after alloactivation (**Figure 6** and data not shown). Furthermore, only CFSE^lo^ alloactivated T cells, which have divided ≥4 times in 48 hours, expressed low but consistently detectable levels of Ceacam1 (**Figure 6A**). These kinetics are consistent with a role for Ceacam1 in regulating early events in T cell alloactivation.

### Ceacam1 regulates the alloactivation and proliferation of T cells

Because the expression of Ceacam1 on alloreactive T cells after adoptive transfer occurred *in vivo* with similar kinetics as T cell alloactivation [30], we asked whether Ceacam1 on either donor alloreactive T cells or radio-resistant cells in allo-BMT recipients could regulate this process. We transferred CFSE-labeled purified B6 WT or Ceacam1^−/−^ splenic T cells into irradiated BALB/c recipients and analyzed donor T cells in spleens on day 3.

We observed that relative to isotype control staining, an increased percentage of alloactivated CFSE^lo^ CD4 Ceacam1^−/−^ T cells were positive for the alloactivation marker CD25, and that a greater percentage of these cells downregulated CD62L than WT T cells (**Figure 6B–C**), suggesting that more of them became activated. Additionally, an increased percentage of donor Ceacam1^−/−^ CD4 T cells had divided to a CFSE^lo^ alloactivated state (**Figure 6D**), suggesting enhanced proliferation in the absence of Ceacam1.

We repeated these experiments with alloreactive Ceacam1-Tg T cells and as expected, observed a decrease in numbers of CFSE^lo^ T cells as assessed by CFSE dilution (**Figure 6E**). This is consistent with an inhibitory role for Ceacam1 in the proliferation of alloreactive T cells. However, we did not observe significant differences in alloactivation between Ceacam1-Tg vs. WT donor T cells (data not shown).

Lastly, we assessed the role of Ceacam1 expression on radio-resistant cells in allo-BMT recipients for donor T cell alloactivation. We transferred CFSE-labeled B6 T cells into irradiated WT vs. Ceacam1^−/−^ BALB/c mice, and analyzed donor T cells in spleens on day 3. Here, we did not observe differences in proliferation (data not shown), but donor CD4 T cells in Ceacam1^−/−^ allogeneic recipients did exhibit an increase in alloactivation as measured by CD25 (**Figure 6F**).

### Ceacam1 does not significantly influence T cell polarization, cytolysis or dendritic cell function in GVHD

We measured serum cytokines in recipients of WT, Ceacam1-Tg and Ceacam1^−/−^ T cells on days 7 and 14 post-transplant, and observed that levels of IFNγ, TNF, IL-2, IL-4, IL-6, IL-10, and IL-12p70 were similar (data not shown). Percentages of FoxP3^+^ donor regulatory T cells and expression of T-bet were also similar between recipients of WT, Ceacam1-Tg and Ceacam1^−/−^ T cells (data not shown and **Table 1**), and stimulation of splenocytes harvested on day 14 after BMT post-transplant from these three groups revealed essentially no IL-17^+^ donor T cells (not shown), and similar percentages of donor IFNγ^+^ T cells (data not shown and **Table 1**).

**Table 1:** Summary of Ceacam1 deficiency or overexpression.

	*Deficient donor T:*	*Transgenic donor T:*	*Deficient recipients:*
Survival	↓	↑	↓
Complete blood count	=	=	=
Engraftment/chimerism	=	=	=
Serum TNF, IFNγ, IL-2, IL-12	=	=	=
Donor T cells			
Activation/Proliferation	↑	↓	↑
T-bet, FoxP3, IFNγ	=	=	=
Trafficking molecules	↑	=	↑
Cytotoxicity	=	n/a	n/a
Numbers in			
Spleen	↑	=	=
MLN/intestine	↑	↓	↑
PLN	↓	↓	↓
Splenic donor DCs			
percentage, apoptosis	=	=	=
Maturation (CD80/86)	=	=	=
Target organ damage			
Liver	=	↓	=
Small intestine	=	↓	=
Large intestine	↑	↓	↑
Thymus	↓	↓	↑

As Ceacam1 can regulate the cytolytic responses of lymphocytes [34], [35], [36], [37], [38], we assessed the cytolytic function of WT vs. Ceacam1^−/−^ alloactivated CD8 T cells from the spleens of allo-BMT recipients on day 14. Ceacam1^−/−^ CD8 T cells and WT CD8 T cells demonstrated similar cytolysis against ^51^Cr-radiolabeled allogeneic A20 B cell lymphoma cells and EL4 controls (**Table 1**). Lastly, we found no differences in DC numbers, activation state (CD80, CD86, MHC class II) from the infusion of Ceacam1^−/−^ or Ceacam1-Tg T cells (**Table 1**), or in Ceacam1^−/−^ allo-BMT recipients.

### Ceacam1^−/−^ donor T cells have enhanced graft-versus-tumor activity towards A20 lymphoma but not renal cell carcinom

**Figure 7:**
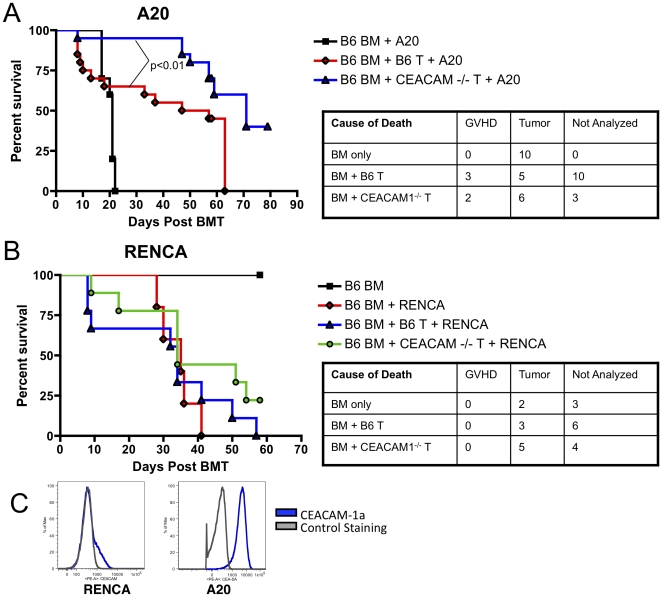
Ceacam1^−/−^ T cells have intact graft-versus-tumor activity. A) 5×10^6^ B6 BM+0.5×10^6^ A20±0.5×10^6^ B6 WT or Ceacam1^−/−^ T cells→BALB/c (8.5 Gy). N = 20/group for groups receiving T cells, combined from two experiments. B) 5×10^6^ B6 BM±0.1×10^6^ RENCA±0.8×10^6^ B6 WT or Ceacam1^−/−^ T cells→BALB/c (8.5 Gy). N = 9 for groups receiving T cells. C) A20 lymphoma cells and RENCA tumor cells in culture were stained for Ceacam1 expression.

Finally, we assessed the GVT activity of Ceacam1^−/−^ donor alloreactive T cells against A20 lymphoma and RENCA renal cell carcinoma. Recipients of Ceacam1^−/−^ donor T cells had improved survival in the A20 lymphoma model (**Figure 7A**), but both T cell replete groups showed comparable survival in the RENCA solid tumor model (**Figure 7B**). When we analyzed these two tumor lines for Ceacam1 expression, we noted that all A20 lymphoma cells uniformly expressed high levels, while only a subset of RENCA cells expressed some Ceacam1 (**Figure 7C**).

## Discussion

In this report, we show that Ceacam1, which is found on both donor alloreactive T cells as well as non-hematopoietic tissues such as gastrointestinal and hepatic epithelium, can regulate both donor T cell function and the sensitivity of allo-BMT recipients to radiation-containing preparative regimens. In addition, Ceacam1 on donor T cells and tumors may modulate GVT activity. Ceacam1 on both the donor allograft and recipient tissues thus appears to represent an important regulator of GVHD and GVT morbidity and mortality via both T cell dependent and independent mechanisms, suggesting that therapeutic approaches which modulate Ceacam1 may need to assess and balance GVHD vs. GVT.

Ceacam1 on T cells has previously been shown to restrain CD4 T cell polarization, cytokine secretion and cytotoxicity. In our GVHD model systems however, we found similar T cell polarization and cytokine secretion when we analyzed donor alloreactive T cells *ex vivo* (**Table 1**). We ascribe this to the strongly proinflammatory cytokine milieu found in recipients following myeloablative radiation treatment, as well as the ubiquitous presence of alloantigen, which together promote strong Th1 differentiation regardless of Ceacam1 expression. However, in our model systems Ceacam1 regulated T cell activation, and numbers of donor alloactivated T cells in both lymphoid tissues and GVHD target tissues, in patterns that generally correlated with levels target organ damage (**Figures 2** **and** **3**).

We also assessed the role of Ceacam1 in allo-BMT recipients. In our model systems, WT T cells in a Ceacam1-deficient environment showed a phenotype similar to that of Ceacam1^−/−^alloactivated T cells: both showed increased activation, selective damage to the large intestines, and preferential accumulation in the MLN and intestinal parenchyma of mice with GVHD, and correspondingly decreased infiltration of the liver and PLN, ultimately leading to exacerbation of disease, with accelerated mortality in the first two weeks post-transplant. This suggests that Ceacam1 on donor T cells interacts with recipient tissues, and that Ceacam1 “fraternal” interactions between cells of the donor graft, were not sufficient to restrain GVHD in Ceacam1^−/−^ recipients. However, the increased early mortality of Ceacam1^−/−^ allo-BMT recipients with GVHD also led us to ask whether Ceacam1^−/−^ mice were sensitive to radiation injury. While Ceacam1^−/−^ mice were not significantly defective for hematopoiesis after sublethal irradiation at 3.5 and 4.5 Gy (data not shown), they did exhibit significantly increased damage to the small intestines after lethal irradiation (**Figure 4**).

Ceacam1 also directly regulates intestinal epithelia. Due to enhanced Wnt/β-catenin signaling, Ceacam1^−/−^ jejunal and ileal enterocytes exhibit higher levels of the positive cell cycle regulators c-Myc and cyclin D1 [41]. Dysregulated c-Myc may sensitize cells to apoptosis [42], [43], and higher levels of these proteins may render Ceacam1^−/−^ enterocytes more sensitive to radiation injury. Finally, Ceacam1 also regulates cell-cell adhesion [17], [18], [44], [45] under normal and pathological conditions; it may therefore also be possible that loss of Ceacam1 regulates radiation-induced sloughing of intestinal epithelium.

It is difficult to directly assess the relative importance of gastrointestinal radiation sensitivity versus increased GVHD in Ceacam1^−/−^ allo-BMT recipients, as radiation-induced gut damage may both be directly manifested in intestinal pathology, yet transmural migration of bacterial superantigens is an important first step for the initiation of GVHD[1], [39], [40], and increased damage to the intestines of Ceacam1^−/−^ mice may thus amplify the development of GVHD in these mice, and also explain in part the specifically increased large intestinal GVHD we observed.

In experiments with Ceacam1^−/−^ donor T cells, we also observed a trend for splenic donor CD8 alloactivated T cells to express higher levels of α_4_β_7_. Although integrin α_4_β_7_ is important for GVHD pathogenesis, and we have previously shown that β_7_^−/−^ T cells cause a sustained decrease in acute systemic and intestinal GVHD [31], differential expression of integrin α_4_β_7_ by Ceacam1^−/−^ T cells is almost certainly only one part of how Ceacam1 regulates target organ GVHD. Indeed, donor alloactivated CD4 T cells expressed comparable levels of integrin α_4_β_7_ as wildtype cells, yet were also found in increased numbers in the gut (**Figure 3A**). This suggests that other mechanisms, such as Ceacam1 regulation of donor T cell activation (**Figure 6**) may also contribute to its regulation of GVHD target organ damage.

Moreover, recipients of Ceacam1-Tg T cells also had reduced intestinal infiltrates despite similar integrin α_4_β_7_ expression, suggesting that Ceacam1 regulates the accumulation of donor T cells in target tissues via multiple mechanisms. Thus, our results on donor lymphocyte infiltrates into GVHD target tissues and secondary lymphoid tissues must be interpreted cautiously, as they must be influenced by T cell proliferation, retention and apoptosis, in addition to trafficking.

Although Ceacam1^−/−^ and Ceacam1-Tg T cells displayed overall symmetric and opposite phenotypes, we also noted differences. Ceacam1-Tg T cells primarily showed decreased proliferation, whereas Ceacam1^−/−^ T cells showed changes in proliferation, but also trafficking and activation. Some of these differences may be due to our models: on WT T cells, Ceacam1 is only briefly and transiently upregulated during activation. Consequently, Ceacam1^−/−^ T cells are “missing” Ceacam1 only transiently, while Ceacam1-Tg T cells constitutively over-express Ceacam1. Furthermore, while Ceacam1^−/−^ T cells are effectively insensitive to all Ceacam1 ligands and interactions, Ceacam1-Tg T cells which over-express the protein may have increased fraternal Ceacam1 interactions with other donor T cells, but may not necessarily experience increased Ceacam1 interactions with donor BM or host hematopoietic and non-hematopoietic components. These differences may explain why their activation and trafficking phenotypes are not directly opposed.

We were interested to note that in our GVT experiments, recipients of Ceacam1^−/−^ T cells had significantly improved survival when challenged with A20 lymphoma but not renal cell carcinoma. Although both A20 lymphoma and renal cell carcinoma express Ceacam1, A20 cells uniformly expressed Ceacam1 at high levels, while only a subset of RENCA cells showed (somewhat lower) expression. Indeed, a number of hematologic tumors, including EL4 leukemia, P815 mastocytoma, and C1498 myeloid leukemia all express substantial levels of Ceacam1 (data not shown), whereas some solid tumors, such as mouse 4T1 breast epithelial cancer and CT51 colon tumor normally express only lower or even minimal levels of Ceacam1, similar to the lower level of expression we found with RENCA (not shown).

Therefore, one possibility is that the GVT activity of T cells can be negatively regulated by tumors expressing high levels of Ceacam1, but is less important for tumors that express low levels or only on a subset of cells in the first place. However, RENCA in our GVT model systems is found primarily in the liver, and to a lesser extent, the lungs. Since donor allografts with Ceacam1^−/−^ T cells showed decreased numbers of donor alloreactive T cells in the liver as compared with wildype in GVHD experiments (**Figure 3A**), interpretation of GVT activity against RENCA with respect to Ceacam1 on T cells must also consider this aspect of its biology.

In conclusion, our results show that Ceacam1 on both donor T cells and allo-BMT recipients controls the proliferation, activation, and trafficking of donor alloreactive T cells, and the sensitivity of gastrointestinal tissues to irradiation. Consequently, Ceacam1 may represent a viable target for reducing radiation-associated gastrointestinal toxicity, for the control of GVHD and GVT activity after allo-BMT.

## Materials and Methods

### Ethics Statement

All animal protocols were approved by the Memorial Sloan-Kettering Cancer Center (MSKCC) Institutional Animal Care and Use Committee (protocol #99-07-025).

### Mice

C57BL/6 (B6, H-2^b^), BALB/c (H-2^d^), and B10.BR (H-2^k^) mice were obtained from The Jackson Laboratory (Bar Harbor, ME). B6 and BALB/c Ceacam1^−/−^ mice, and B6 Ceacam1-Tg mice were generated at McGill University (B6 and BALB/c from Harlan (Montreal, Quebec, Canada)), and maintained at Memorial Sloan-Kettering Cancer Center. Mice used were between 8 and 12 weeks old.

### Bone Marrow Transplantation

BM cells removed from femurs and tibias were T cell-depleted (TCD) with anti-Thy-1.2 and low-TOX-M rabbit complement (Cedarlane Laboratories, Hornby, ON, Canada). Enriched splenic T cells were obtained by nylon wool column passage. Cells were resuspended in DMEM and injected into lethally irradiated recipients on day 0 after total body irradiation (^137^Cs source) as a split dose 3 hours apart.

### T cell carboxyfluorescein diacetate succinimidyl ester (CFSE) labeling and transfer

Purified splenic T cells were incubated with CFSE (Invitrogen, Carlsbad, CA) at a concentration of 2.5–5 µM in PBS (5×10^7^ cells/mL) at 37°C for 20 minutes, washed twice with PBS, resuspended in DMEM and infused intravenously into lethally irradiated allogeneic recipients. Splenocytes from recipients were harvested at varying time points and analyzed by FACS as described.

### Assessment of GVHD

Survival was monitored daily, and mice were scored weekly for 5 clinical parameters (weight, posture, activity level, fur ruffling, and skin lesions) on a scale from 0 to 2. A clinical GVHD score was generated by summation of the 5 criteria scores; mice scoring 5 or greater were considered moribund and euthanized.

### Histopathologic analysis

Small and large bowel, liver, and skin were assessed by experts in a blinded fashion. Organs were preserved in formalin, transferred to 70% ethanol, and then embedded in paraffin, sectioned, stained with hematoxylin and eosin, and scored with a semi-quantitative scoring system. Bowel and liver were scored for 19 to 22 different parameters associated with GVHD (detailed in **Table S1**); skin was evaluated for number of apoptotic cells/mm^2^ of epidermis via terminal deoxynucleotide transferase dUTP nick end labeling (TUNEL).

### Statistical analysis

Histopathologic scores, median fluorescence intensities and cell counts were compared between groups using the nonparametric unpaired Mann-Whitney U test; the Mantel-Cox log-rank test was used for survival data.

### Additional methods

Additional methods are described in **Methods S1**.

## Supporting Information

Figure S1 **Generation of Ceacam-1 transgenic mice and expression of Ceacam1 on transgenic T cells.** A) The CC1-4L cDNA, expressing 4 Ig domains and the long cytoplasmic domain was inserted into the unique EcoR1 site within the VAhCD2 vector containing the hCD2 promoter and 2 polyadenylation sites (PolyA1,2). B) The linearized construct was microinjected into C57Bl/6 oocytes to produce transgenic mice that were identified by Southern blot with a 1.3 kb 32P-labelled probe. This probe cross-reacts with the endogenous Ceacam1, Ceacam2 and Ceacam10 genes and also identifies the 1.7 kb EcoR1-digested transgene. C) Tg mice were identified by PCR amplification of a 320 bp fragment from tail genomic DNA with the CyT2 oligo within the CC1-L cytoplasmic domain and oligo CD2A2 within the hCD2 LCR region. D) Western blots of lysates from thymi and spleens from 5 and 8-week-old WT and Tg littermates with the rabbit polyclonal anti-Ceacam1 Ab 2457. E–F) Cell surface Ceacam1 on thymic (E) and splenic (F) CD3-gated T cells of WT and Tg mice (n = 4) was revealed with anti-Ceacam1 Ab 2457. Controls were normal rabbit serum.
(TIFF)

Figure S2 **Ceacam1^−/−^ mice exhibit increased thymic and splenic cellularity compared with wildtype animals, but do not exhibit skewing towards particular leukocyte lineages or subsets, while Ceacam1-Tg transgenic mice have similar numbers of splenocytes, thymocytes, and bone marrow cells compared with WT animals.** A) 2 month old Ceacam1^−/−^ male BALB/c mice were analyzed with age and sex-matched wildtype BALB/c, N = 5. Similar results were observed with age and sex-matched female wildtype and Ceacam1^−/−^ BALB/c mice (N = 5, not shown), and in age and sex-matched wildtype and Ceacam1^−/−^ B6 mice (N = 5, not shown). B) Thymocytes from mice in (A) were analyzed by flow cytometry. N = 5. C) 8-week old B6 and Ceacam1-Tg mice were analyzed for splenic, thymic and bone marrow (BM) cellularity. N = 3/group.(TIFF)

Table S1 **Histopathological scoring scheme for gastrointestinal GVHD target organs.**
(DOC)

Methods S1 (DOC)
