# Supplementary figures and images for: Ceacam1 Separates Graft-versus-Host-Disease from Graft-versus-Tumor Activity after Experimental Allogeneic Bone Marrow Transplantation
Source: PLoS One. 2011 Jul 6;6(7):e21611. doi: 10.1371/journal.pone.0021611 (PMC3130781; doi:10.1371/journal.pone.0021611)

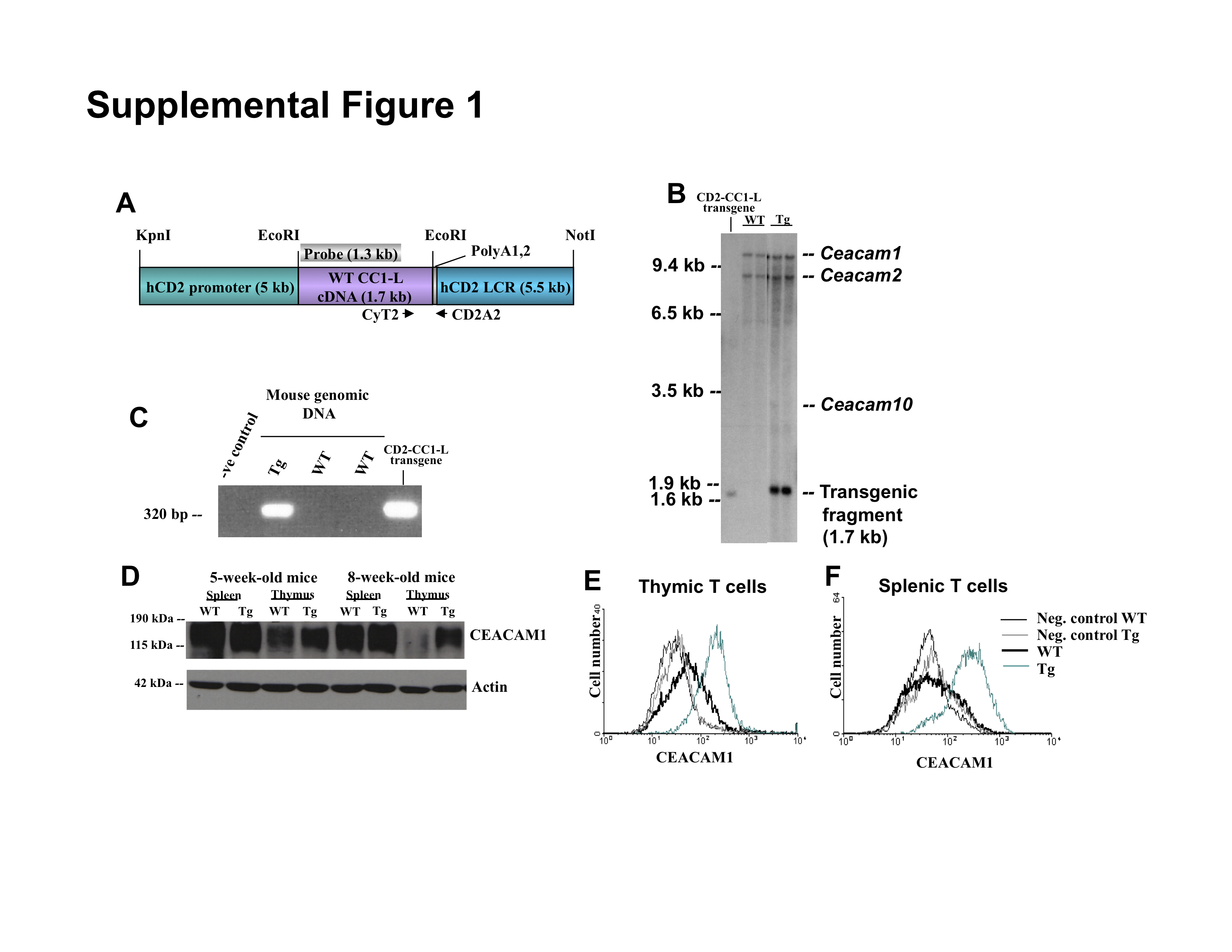

Supplement: Figure S1 — Generation of Ceacam-1 transgenic mice and expression of Ceacam1 on transgenic T cells. A) The CC1-4L cDNA, expressing 4 Ig domains and the long cytoplasmic domain was inserted into the unique EcoR1 site within the VAhCD2 vector containing the hCD2 promoter and 2 polyadenylation sites (PolyA1,2). B) The linearized construct was microinjected into C57Bl/6 oocytes to produce transgenic mice that were identified by Southern blot with a 1.3 kb 32P-labelled probe. This probe cross-reacts with the endogenous Ceacam1, Ceacam2 and Ceacam10 genes and also identifies the 1.7 kb EcoR1-digested transgene. C) Tg mice were identified by PCR amplification of a 320 bp fragment from tail genomic DNA with the CyT2 oligo within the CC1-L cytoplasmic domain and oligo CD2A2 within the hCD2 LCR region. D) Western blots of lysates from thymi and spleens from 5 and 8-week-old WT and Tg littermates with the rabbit polyclonal anti-Ceacam1 Ab 2457. E–F) Cell surface Ceacam1 on thymic (E) and splenic (F) CD3-gated T cells of WT and Tg mice (n = 4) was revealed with anti-Ceacam1 Ab 2457. Controls were normal rabbit serum. (TIFF) [file pone.0021611.s001.tif]

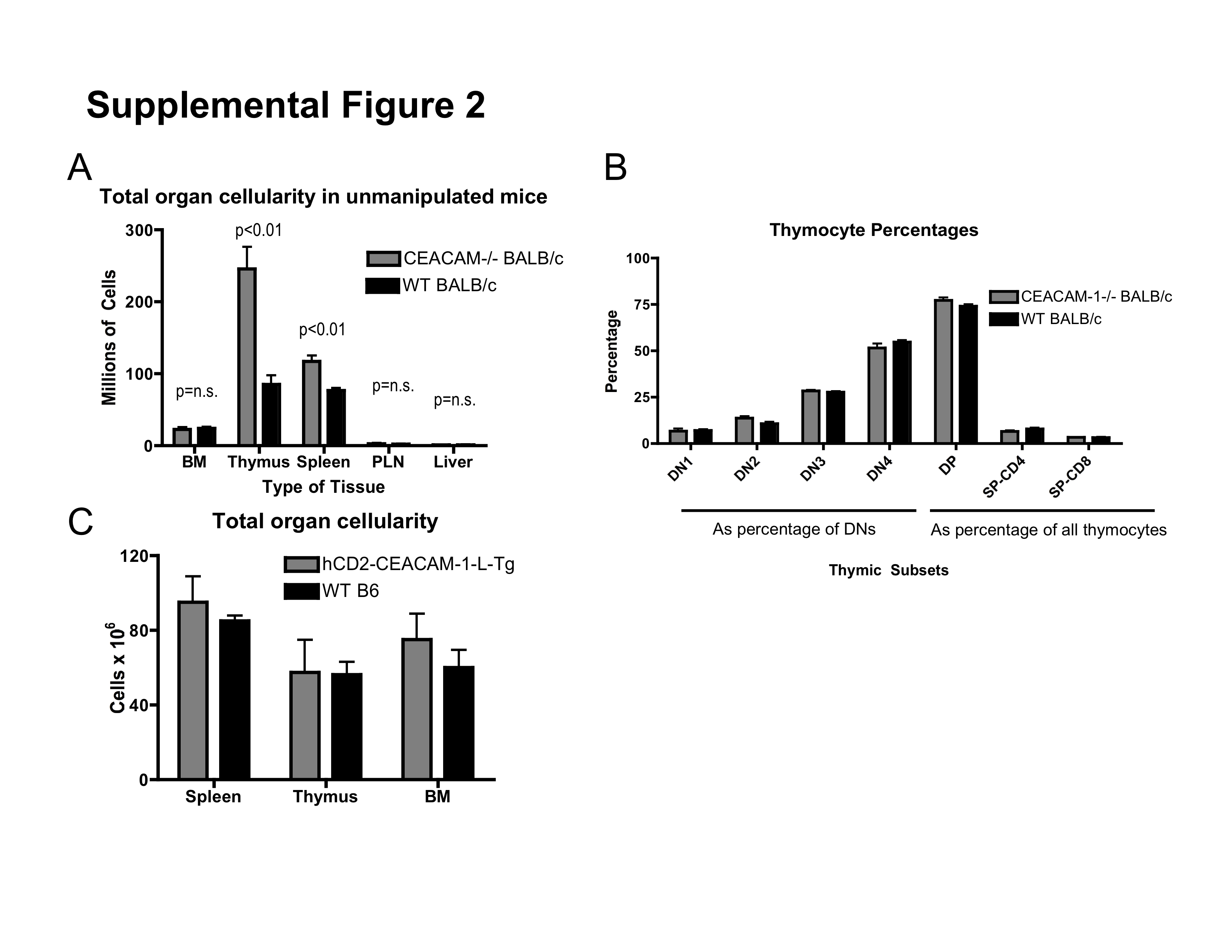

Supplement: Figure S2 — Ceacam1−/− mice exhibit increased thymic and splenic cellularity compared with wildtype animals, but do not exhibit skewing towards particular leukocyte lineages or subsets, while Ceacam1-Tg transgenic mice have similar numbers of splenocytes, thymocytes, and bone marrow cells compared with WT animals. A) 2 month old Ceacam1−/− male BALB/c mice were analyzed with age and sex-matched wildtype BALB/c, N = 5. Similar results were observed with age and sex-matched female wildtype and Ceacam1−/− BALB/c mice (N = 5, not shown), and in age and sex-matched wildtype and Ceacam1−/− B6 mice (N = 5, not shown). B) Thymocytes from mice in (A) were analyzed by flow cytometry. N = 5. C) 8-week old B6 and Ceacam1-Tg mice were analyzed for splenic, thymic and bone marrow (BM) cellularity. N = 3/group.(TIFF) [file pone.0021611.s002.tif]
