# Supplementary material for: Ceacam1 Separates Graft-versus-Host-Disease from Graft-versus-Tumor Activity after Experimental Allogeneic Bone Marrow Transplantation
Source: PLoS One. 2011 Jul 6;6(7):e21611. doi: 10.1371/journal.pone.0021611 (PMC3130781; doi:10.1371/journal.pone.0021611)
Supplement: Table S1 — Histopathological scoring scheme for gastrointestinal GVHD target organs. (DOC) [file pone.0021611.s003.doc]

| **Table S1: histopathological scoring scheme for gastrointestinal GVHD target organs** | | |
| --- | --- | --- |
| **Liver Features** | **Small Bowel Features** | **Large Bowel Features** |
| *Portal Triads:* | *Architecture:* | *Architecture:* |
| Portal Tract expansion | Villus blunting | Crypt regeneration |
| Neutrophil infiltrate | Crypt regeneration | Surface erosion |
| Mononuclear infiltrate | Surface erosion | Ulceration |
| *Bile Ducts/Ductules:* | Ulceration | Lamina propia inflammation |
| Mononuclear infiltrate of epithelium | Lamina propia inflammation | Atrophy (chronic) |
| Nuclear pleomorphism | Atrophy (chronic) | Crypt branching (chronic) |
| Cytoplasmic eosinophilia | Endocrine cell excess (chronic) | Endocrine cell excess (chronic) |
| Nuclear multilayering | Paneth cell excess (chronic) | Paneth cell excess (chronic) |
| Pyknotic duct cells | *Epithelial Cytology:* | *Epithelial Cytology:* |
| Intraluminal epithelial cells | Vacuolization | Vacuolization |
| Other intraluminal cells | Loss of microvillus brush border | Attenuation (from columnar) |
| *Vascular:* | Attenuation (from columnar) | Apoptosis |
| Endothelialitis | Apoptosis | Sloughing into lumen |
| Mononulcear cells around CV | Sloughing into lumen | Lymphocytic infiltrate |
| *Hepatocellular Damage:* | Lymphocytic infiltrate | Neutrophilic infiltrate |
| Confluent necrosis | Neutrophilic infiltrate | *Vascular:* |
| Acidophilic bodies | *Vascular:* | Edema |
| Mitotic figures | Edema | Hemorrhage |
| Foamy change | Hemorrhage | Granulation tissue |
| Ballooning degeneration | Granulation tissue | Hemosiderin (old damage) |
| Neutrophil accumulations | Hemosiderin (old damage) |  |
| Macrophage aggregates |  |  |
| Macrocytosis (regeneration) |  |  |
| Extramedullary hematopoiesis |  |  |
