# Supplementary material for: Ceacam1 Separates Graft-versus-Host-Disease from Graft-versus-Tumor Activity after Experimental Allogeneic Bone Marrow Transplantation
Source: PLoS One. 2011 Jul 6;6(7):e21611. doi: 10.1371/journal.pone.0021611 (PMC3130781; doi:10.1371/journal.pone.0021611)
Supplement: Methods S1 — (DOC) [file pone.0021611.s004.doc]

**METHODS S1**

**Flow cytometry**

Lymphocytes were incubated for 10 minutes at 4°C with anti-CD16/CD32 FcR block and stained with fluorochrome-labeled antibodies at saturating concentrations for 15-30 minutes at 4°C. Flow cytometric analysis was performed on a LSR II (Becton Dickinson, San Jose, CA). Data analysis was performed with FlowJo 8 (Treestar, San Carlos, CA) and Excel (Microsoft, Redmond, WA).

**Antibodies**

Rat anti-mouse CD16/CD32 FcR block (2.4G2) and fluorochrome-labeled antimurine antibodies against CD3ε (145-2C11), CD4 (RM4-5), CD8 (53-6.7), CD62L (MEL-14, CD44 (IM7), CD25 (PC61), CD45.1 (A20), H-2K^b^ (AF6-88.5), CD28 (37.51), and strepavidin PE-TexasRed were obtained from BD Pharmingen (San Diego, CA). CD44 (IM7) was obtained from Biolegend (San Diego, CA). FoxP3 antibody was obtained from eBioscience (San Diego, CA). CC1 anti-Ceacam1 antibody has been described previously(26) and was purified and biotin-conjugated by the Memorial Sloan-Kettering Cancer Center monoclonal antibody core facility. The Ceacam1 polyclonal antibody 2457 was generated at McGill University.

**Intracellular staining for cytokines and transcription factors**

To detect FoxP3 and T-bet expression, lymphocytes were stained with cell surface antibodies, fixed with Fix/Perm buffer from eBioscience (San Diego, CA) for 30 minutes, washed and then stained in permeabilization buffer with intracellular antibodies for 30 minutes, washed in permeabilization buffer and resuspended in PBS for flow cytometry. In some experiments, cytokine secretion was inhibited with Brefeldin A or Golgiplug™ from BD Biosciences (San Diego, CA).

**Cytotoxicity assay**

Target cells were labeled with 100 µCi (3.7 MBq) of ^51^Cr at 3 x 10^6^ cells/mL for 1 hour at 37°C and plated at 2.5 x 10^3^ cells/well in 96-well U bottom plates (Costar, Cambridge, MA). Splenocytes from mice 14 days after HSCT with allografts consisting of WT bone marrow and either WT or CEACAM1^-/-^ T cells were analyzed by FACS for donor markers and CD3/CD8 purity and were added at various effector-target ratios in a final volume of 200 µL in triplicate and incubated for 4 hours at 37°C. Subsequently, 35 µL supernatant was removed from each well and was counted in a gamma counter (Topcount-Packard, Meriden, CT) to determine experimental release. Spontaneous release was obtained from wells receiving target cells with medium, and total release was obtained from wells receiving 5% Triton X-100. Percentage cytotoxicity was calculated as follows: percentage cytotoxicity = 100 x (experimental release - spontaneous release)/(total release - spontaneous release).

**Cytometric Bead Array Assay**

Sera were collected and stored at -80°C until analysis. Levels of TNFα, IFNγ, IL-2, IL-4, IL-5, IL-6, IL-10, IL-12p70 and MCP-1 were determined by Cytometric Bead Array (Becton Dickson, San Diego, CA) per manufacturer's instructions.

**Lymphocyte isolation from liver and gut**

Small intestines were flushed with 10% HEPES (N-2-hydroxyethylpiperazine-N '-2-ethanesulfonic acid) + 10% FBS, cut into 1-cm-long pieces, and incubated for 1 hour at 37° C with continuous shaking. Intestinal pieces were then vortexed for 15 seconds, and the supernatant was strained and centrifuged at 325 g for 5 minutes.

Livers were homogenized and passed through a 70-µm cell strainer. Pellets were resuspended in 40% Percoll and layered above 70% Percoll and centrifuged to obtain lymphocytes.

**Enumeration of donor lymphocytes from target tissues**

Spleens, lymph nodes, and Peyer’s patches were dissected from mice and a single-cell suspension produced by mashing between frosted glass slides. An enriched preparation of non-parencymal cells from liver and small intestine (for intraepithelial lymphocytes) was prepared as described above. We identified viable donor hematopoietic cells as CD45^+^DAPI^dim^H-2Kb^+^. In some experiments, the donor/host marker was CD45.1. Donor T cells were identified as CD3ε^+^CD4^+^ or CD3ε^+^CD8^+^.

**Cell lines**

Renal cell carcinoma (RENCA; H-2^d^), EL4 leukemia (H-2^b^), and A20 lymphoma (H-2^d^) were cultured in RPMI 1640, 10% heat-inactivated FBS, 100 U/ml penicillin, 100 mg/ml streptomycin and 2 mM L-glutamine.
